# Upcycling of Wastewater via Effective Photocatalytic Hydrogen Production Using MnO_2_ Nanoparticles—Decorated Activated Carbon Nanoflakes

**DOI:** 10.3390/nano10081610

**Published:** 2020-08-17

**Authors:** Sankar Sekar, Sejoon Lee, Preethi Vijayarengan, Kaliyappan Mohan Kalirajan, Thirumavalavan Santhakumar, Saravanan Sekar, Sutha Sadhasivam

**Affiliations:** 1 Division of Physics & Semiconductor Science, Dongguk University-Seoul, Seoul 04620, Korea; sanssekar@gmail.com; 2 Quantum-Functional Semiconductor Research Center, Dongguk University-Seoul, Seoul 04620, Korea; 3 Renewable Energy Lab, Hindustan Institute of Technology and Science, Chennai 603103, Tamil Nadu, India; santhakumarbecivil@gmail.com; 4 Department of Nanotechnology, K.S.R College of Technology, Tiruchengode 637215, Tamil Nadu, India; tony85kali@gmail.com; 5 Department of Mechanical Engineering, K. Ramakrishnan College of Technology, Trichy 621112, Tamil Nadu, India; nanosaran007@gmail.com; 6 Department of Chemistry, Periyar University, Salem 636011, Tamil Nadu, India; suthaasridhar@gmail.com

**Keywords:** manganese oxide, activated carbon, nanocomposite, photocatalyst, green synthesis, hydrogen production

## Abstract

In the present work, we demonstrated the upcycling technique of effective wastewater treatment via photocatalytic hydrogen production by using the nanocomposites of manganese oxide-decorated activated carbon (MnO_2_-AC). The nanocomposites were sonochemically synthesized in pure water by utilizing MnO_2_ nanoparticles and AC nanoflakes that had been prepared through green routes using the extracts of Brassica oleracea and Azadirachta indica, respectively. MnO_2_-AC nanocomposites were confirmed to exist in the form of nanopebbles with a high specific surface area of ~109 m^2^/g. When using the MnO_2_-AC nanocomposites as a photocatalyst for the wastewater treatment, they exhibited highly efficient hydrogen production activity. Namely, the high hydrogen production rate (395 mL/h) was achieved when splitting the synthetic sulphide effluent (S^2−^ = 0.2 M) via the photocatalytic reaction by using MnO_2_-AC. The results stand for the excellent energy-conversion capability of the MnO_2_-AC nanocomposites, particularly, for photocatalytic splitting of hydrogen from sulphide wastewater.

## 1. Introduction

Upcycling of wastewater via effective solar-assisted photocatalytic hydrogen production is vital for future green energy technology because this technique would become readily available as an ecofriendly and inexpensive method for treating industrial wastewater as well as developing alternative energy sources. Among various attractive methods for water splitting (i.e., hydrogen production), photocatalysis is one of the emerging techniques for the development of energy conversion technology [1,2,3]. For photocatalytic hydrogen production, developing a novel semiconductive material is essential to enhance the charge separation because the large number of photo-generated charge carriers may promote the photocatalysis reaction [4]. In general, semiconductor materials exhibit the increased potential energy from the photocatalytic reaction because of their flexible constitution and diversity of properties [5]. Even though a variety of inorganic semiconductors showed a potential hydrogen production ability through photocatalytic reactions, there has been a serious drawback in water-splitting due to its absorption of visible light and bandgap variations [6]. As an alternative, transition metal oxides have become a prominent candidate for photocatalytic hydrogen production because of their modulated interface, tunable bandgap, high specific surface area, and good electron transport [7,8]. Among various metal oxides, MnO_2_ acts as a diverse material in technical and fundamental aspects. For example, MnO_2_ exhibited various feasibilities in wide application areas; e.g., catalysis, molecular adsorption, sensors, energy storage electrodes, etc. [9,10,11,12,13]. In addition, MnO_2_ can also be available for environmental technology such as dye degradation [14,15], wastewater treatment [16,17], photocatalytic degradation of organic pollutants [18,19,20,21,22,23], and photo-electrochemical hydrogen production [24,25].

To move a step closer to feasible applications, recently, intensive research was conducted to synthesize high-quality MnO_2_ [26,27]. However, most of the well-known synthesis techniques are based on chemical approaches, which generally require toxic and/or hazardous precursors [28]. To release this, many researchers have been devoted to developing a green synthesis method, particularly, using biomass natural resources (e.g., plant and fruit extracts) as chemical reagents [29,30]. For instance, the ecofriendly-synthesized MnO_2_ nanoparticles showed similar structural and morphological properties to the chemically synthesized ones [31,32]. Furthermore, those MnO_2_ nanoparticles could be utilized in catalysis because of their excellent physicochemical properties [33]. Meanwhile, adding activated carbon (AC) into catalysts is also of great interest for improving the photocatalytic reaction. In other words, since low-cost AC has several advantages (e.g., high porosity, specific surface area, and excellent adsorption), catalytic performances could be improved during photocatalytic hydrogen production [34]. Additionally, many earlier studies reported that biomass AC could improve the catalytic ability for reformation of carbon dioxide [35] and enhance the energy-storage performances [36,37,38,39]. Despite such vast benefits, to our best awareness, no studies on the MnO_2_-AC nanocomposite-based wastewater treatment have been conducted yet. We, therefore, investigated the formation of high-performance MnO_2_-AC photocatalysts via ecofriendly routes for efficient hydrogen production.

Herein, we report experimental data on the effective photocatalytic hydrogen production from wastewater using a novel nanocomposite of the MnO_2_-AC photocatalyst that had been prepared by green approaches. The MnO_2_-AC nanocomposites were fabricated through a simple sonication process in pure water by using MnO_2_ nanoparticles and AC nanoflakes that were synthesized using *Brassica oleracea* (cabbage) and *Azadirachta indica* (neem leaf) extracts, respectively. We observed the high photocatalytic activity of the MnO_2_-AC nanocomposites, resulting from the increase in photo-generated electron and hole carriers in the composite system. In this article, the material preparation and the improved hydrogen production characteristics of the MnO_2_-AC nanocomposites are discussed in detail.

## 2. Experimental Details

### 2.1. Synthesis of MnO_2_ Nanoparticles

**Figure 1:**
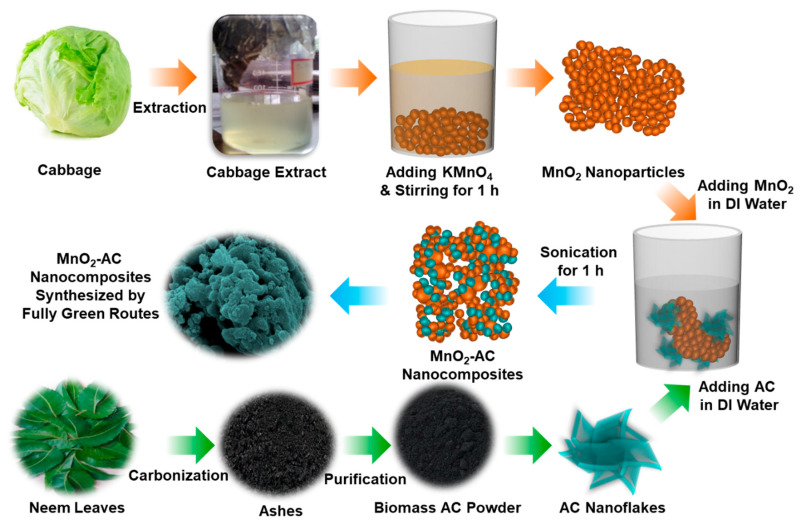
Schematic illustration for the fabrication of MnO_2_ nanoparticles, AC nanoflakes, and MnO_2_-AC nanocomposites.

Figure 1 schematically illustrates the experimental procedure for the synthesis of the MnO_2_-AC nanocomposites. To synthesize the composite structure of MnO_2_-AC, as a primary task, we prepared the MnO_2_ nanoparticles by reducing potassium permanganate (KMnO_4_: 99.7%, Sigma-Aldrich, St Louis, MO, USA) using the reduction agent of *Brassica oleracea*, which had been extracted from cabbage (Perambalur, Tamil Nadu, India). Firstly, 0.1 M of KMnO_4_ (30 mL) was mixed with 50 mL of *Brassica oleracea* in dropwise, and the mixture solution was left for 15 min. Thereafter, the brownish colloidal suspension was stirred at 600 rpm for 1 h by using a home-built magnetic stirrer. During this step, molecular constituents (e.g., anthocyanins and cyanides) of *Brassica oleracea* act as effective reduction agents [40,41] to reduce KMnO_4_ into the form of nanocrystalline MnO_2_. The obtained product was then washed by deionized (DI) water and an ethanol–water mixture to remove both template extracts and unreacted chemicals. After further washing, filtering, drying, and grinding, the powder type of MnO_2_ nanoparticles were obtained.

### 2.2. Derivation of Biomass AC

Next, we derived the biomass AC powders by using *Azadirachta indica* (neem leaves) that had been collected from Perambalur, Tamil Nadu, India. Firstly, the neem leaves were washed and dried several times to get clean *Azadirachta indica.* Then, the dried leaves were carbonized at 500 °C for 2 h. Subsequently, the carbonized ashes were purified through the hydrolysis treatment using DI water to increase the uniformity of the AC nanoflakes [37,38]. After drying the water-treated ashes at 120 °C for 10 h, filtering and grinding processes were performed to obtain the uniform AC nanopowders.

### 2.3. Synthesis of MnO_2_-AC Nanocomposites

Using the above materials, we synthesized the MnO_2_-AC nanocomposites. Firstly, MnO_2_ nanoparticles and AC nanoflakes were mixed in DI water with the ratio of 1:1. Next, to form the MnO_2_-AC nanocomposites, the mixture solution was then sonicated by using the UD-211 ultrasonic disruptor (Tomy Digital Biology Co., Tokyo, Japan) under ultrasonic power of 100 W for 1 h at 20 kHz. After sonication, the solution was dried at 120 °C for 12 h to get the powder type of the MnO_2_-AC nanocomposites. Finally, the powders were grinded and filtered to obtain free-flow fine MnO_2_-AC nanoparticles.

### 2.4. Characterization of Material Properties

The crystallographic properties of the MnO_2_ nanoparticles and the MnO_2_-AC nanocomposites were investigated by X-ray diffraction (XRD) using a Rigaku Miniflex 300 system (Rigaku, Tokyo, Japan). The functional groups of MnO_2_ and MnO_2_-AC were recognized by Fourier transform infrared (FTIR) spectroscopy using a Spectrum-100 system (Perkin Elmer, Shelton, CT, USA). The surface area and the pore characteristics were analyzed through Barrett–Joyner–Halenda (BJH) and Brunauer–Emmett–Teller (BET) methods by using a BELSORP-mini II equipment (MicrotracBEL, Osaka, Japan). The topographical and the compositional properties were examined by field-emission scanning electron microscopy (FE-SEM) and in-situ energy dispersive X-ray (EDX) spectroscopy using a SIGMA-VP system (Zeiss, Jena, Germany). The optical properties were evaluated by UV-VIS spectroscopy using an S-3100 system (Scinco, Seoul, Republic of Korea).

### 2.5. Measurement of Photocatalytic Performances

The photocatalytic performances of MnO_2_ and MnO_2_-AC were characterized via direct solar irradiation at Chennai, India. The optical power of the incident sunlight was measured by using a LX-101A lux meter (HTC Instruments, Mumbai, India) at the beginning and end of each experiment. All the experiments were conducted for 1 h during 12 pm to 2 pm to avail the maximum solar irradiation. The average solar irradiance was found to be 725 W/m^2^. For photocatalytic hydrogen production, firstly, the prepared photocatalyst was dispersed in a photolytic solution, containing synthetic sulphide wastewater (1 L). The effects of the sulphide ion (0.05–0.30 M), sulphite ion (0.05–0.30 M), and photocatalyst concentrations (0.1 to 0.3 g/L) on the maximum hydrogen production were examined. The photocatalytic hydrogen production was undertaken in a trapezoidal photo-reactor (5 L), and the amount of hydrogen production was measured by using an inverted measuring cylinder (Figure S1). The photocatalyst is kept under suspension with the help of the recirculation mode by using a peristaltic pump.

## 3. Results and Discussion

### 3.1. Topographical and Compositional Properties

**Figure 2:**
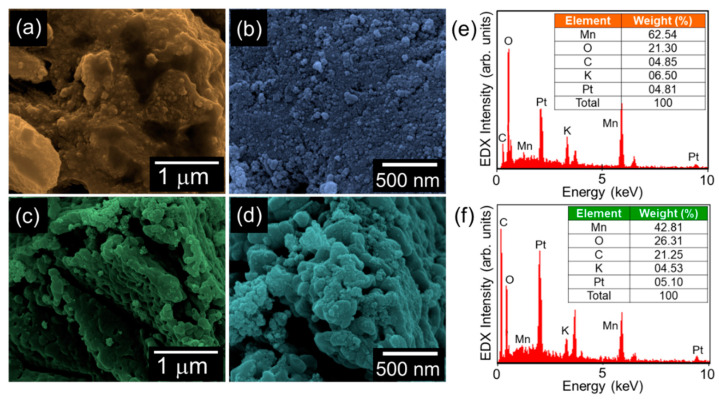
(**a**) Low- and (**b**) high-magnification FE-SEM images of MnO_2_ nanoparticles. (**c**) Low- and (**d**) high-magnification FE-SEM images of MnO_2_-AC nanocomposites. EDX spectra of (**e**) MnO_2_ nanoparticles and (**f**) MnO_2_-AC nanocomposites.

**Figure 3:**
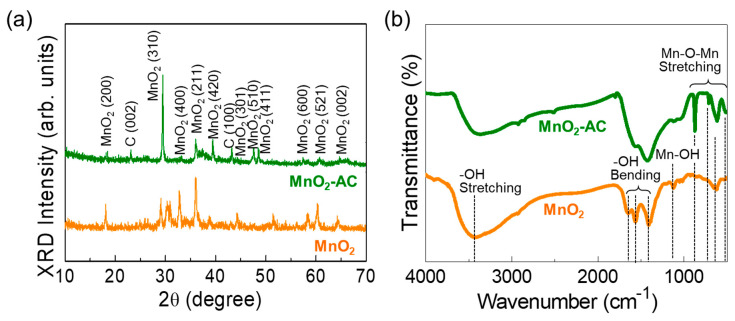
(**a**) XRD patterns and (**b**) FTIR spectra of MnO_2_ nanoparticles and MnO_2_-AC nanocomposites.

**Figure 4:**
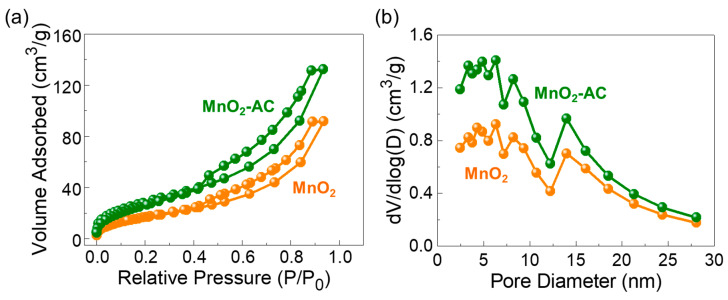
(**a**) Nitrogen adsorption–desorption isotherm characteristics and (**b**) Pore distribution properties of MnO_2_ nanoparticles and MnO_2_-AC nanocomposites.

**Figure 5:**
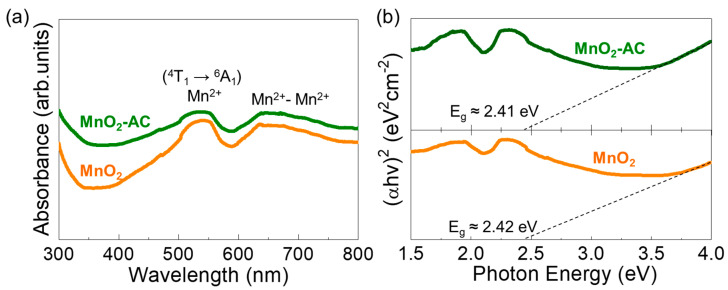
(**a**) UV-VIS absorption spectra and (**b**) Tauc’s plots of MnO_2_ nanoparticles and MnO_2_-AC nanocomposites.

The topographical and the compositional properties of the synthesized materials were investigated by FE-SEM and in-situ EDX, respectively. In the case of bare MnO_2_, a bundle of spherical nanoparticles was aggregated with the shape of the nanogravel field (Figure 2a,b). Similarly, the MnO_2_-AC nanocomposites exhibited an agglomerated topography, where the tiny MnO_2_ nanoparticles were decorated onto the AC nanoflakes (Figure 2c,d). From the EDX spectra (Figure 2e,f), one can confirm that both the MnO_2_ nanoparticles and the MnO_2_-AC nanocomposites contain their main species of Mn, O, and C. Small amounts of Pt and K came from conductive coating for SEM measurements and residual K ions intercalated at MnO_2_ surfaces, respectively. Although the K ions may somewhat affect the hydrogen production efficiency [42,43], we believe that the effect of residual K ions would be negligible because the small amount of K ions were detected only in EDX spectrum but not in other material characterizations, as can be seen from Figure 3, Figure 4 and Figure 5.

### 3.2. Crystallographic Properties

Figure 3a displays the XRD patterns of MnO_2_ and MnO_2_-AC. The MnO_2_ nanoparticles exhibited the diffraction patterns at 18.5, 29.5, 33.0, 36.0, 39.3, 44.2, 47.5, 48.6, 57.3, 60.7, and 64.6°, corresponding to (200), (310), (400), (211), (420), (301), (510), (411), (600), (521), and (002) lattice phases of tetragonal α-MnO_2_ (JCPDS no. 44-0141), respectively [28,39,44]. In the composite of MnO_2_-AC, two additional peaks from C (002) and C (100) phases were observed at 23.2 and 43.2° because of the agglomeration of AC and α-MnO_2_ [36,37,38,45,46]. Using Scherer’s law [47,48,49], the average crystalline size of the MnO_2_ and MnO_2_-AC were calculated to be approximately 32 nm and 28 nm, respectively.

The chemical bonding states of the samples were further elucidated through the FTIR measurements. The MnO_2_ nanoparticles revealed their major IR absorption bands (Figure 3b). The absorption band at 3419 cm^−1^ originates from –OH stretching [50], the vibrational modes at 1643, 1565, and 1419 cm^−1^ arise from –OH bending [51,52], and the IR absorbance at 1121 cm^−1^ comes from the Mn–OH vibration. In addition, MnO_2_ showed to involve some vibration modes at 503, 616, 710, and 868 cm^−1^, arising from Mn–O–Mn stretching in α-MnO_2_ [28,44,51]. In the MnO_2_-AC composites, those Mn–O–Mn stretching modes became significant because the adsorbed C atoms might increase the ionic interaction at the MnO_2_ surface [53,54]. These verify that MnO_2_-AC was well-aggregated with the stable conformation of the composite system.

### 3.3. Textural Characteristics

Figure 4a displays the N_2_ adsorption–desorption isotherm curves of the MnO_2_ nanoparticles and the MnO_2_-AC nanocomposites. All three samples reveal the Type-IV isotherm characteristics with the distinctive Type-H3 hysteresis curves (classified by IUPAC). These are indicative of the mesoporous feature in the solid-state material system [38,39,55,56]. Through the BET analysis, the specific surface area (A_ss_) was determined to be 64, 822, and 109 m^2^/g for MnO_2_, AC (see also Figure S2a), and MnO_2_-AC, respectively. Figure 4b shows the pore characteristics of MnO_2_ and MnO_2_-AC. Both samples clearly revealed their pore sizes in a nanometer scale; i.e., the average pore sizes of MnO_2_ and MnO_2_-AC were estimated to be approximately 8.78 and 7.66 nm, respectively. Through the BJH analysis, additionally, we confirmed that the MnO_2_-AC nanocomposites have a larger total pore volume (i.e., 0.0204 cm^3^/g) than that of the MnO_2_ nanoparticles (i.e., 0.0141 cm^3^/g). In addition, the average pore size and total pore volume of the AC (Figure S2b) are 2.8 nm and 0.0459 cm^3^/g, respectively. The lager pore volume of MnO_2_-AC is thought as resulting from anchoring of mesoporous MnO_2_ nanoparticles with mesoporous AC nanoflakes, and is beneficial for enhancing the photocatalytic hydrogen production, as discussed in detail later.

### 3.4. Optical Properties

The optical properties of the MnO_2_ nanoparticles and the MnO_2_-AC nanocomposites were characterized by means of the Schuster–Kubelka–Munk (SKM) model [57,58] through UV–VIS absorption measurements. Both samples showed the optical absorption bands at the visible wavelength regions (Figure 5a). Namely, the d–d transitions of Mn ions were observed at around 500–550 nm and 620–680 nm. The former is ascribed to the intra-band transition from Mn^2+^ core shells via ^4^T_1_ → ^6^A_1_ [59,60,61], and the latter is attributed to emission from the Mn dimers (i.e., Mn^2+^-Mn^2+^) [62,63]. The photon energy values for those absorption bands can be confirmed to be approximately 2.34 and 1.90 eV, respectively, as represented in Figure 5b. From Tauc’s plots, additionally, the optical bandgap (E*_g_*) was extrapolated to be approximately 2.42 and 2.41 eV for MnO_2_ and MnO_2_-AC, respectively, and those are consistent with the literature value [64].

### 3.5. Photocatalytic Hydrogen Production Efficiencies

**Figure 6:**
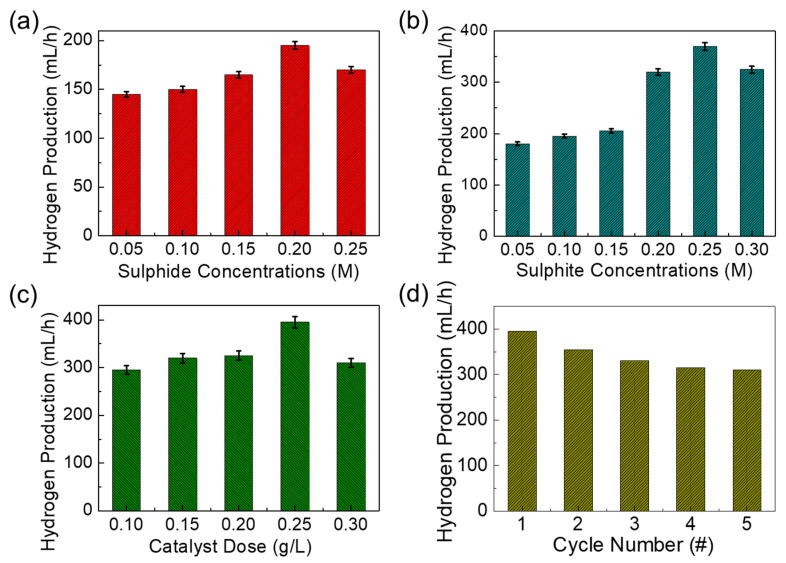
Hydrogen production rates of MnO_2_-AC nanocomposites as functions of (**a**) sulphite ion concentration (best value 0.2 M), (**b**) sulphide ion concentration (best value 0.25 M), (**c**) catalyst dose (best value 0.25 g/L), and (**d**) cycle number of photocatalytic reaction.

Since the bandgap and the absorption energy values of both mesoporous MnO_2_ and MnO_2_-AC are within a photon energy spectrum of the natural sunlight, we assessed their photocatalytic hydrogen production efficiencies using sulphide-based wastewater under sunlight illumination. Here, it should be noted that we varied the sulphide and the sulphite ion concentrations from 0.05 to 0.30 M to examine the effects of sulphur concentrations on photocatalytic hydrogen production. Firstly, we explain the photocatalytic hydrogen-production activity of the MnO_2_-AC nanocomposites for the sulphide ion-added wastewater treatment. To make a similar condition to industrial sulphide wastewater, we prepared synthetic sulphide wastewater by mixing sodium sulphide salt into DI water. In addition, 0.1 M sulphite ion was also added as a sacrificial agent to avoid the photo corrosion of the photocatalyst. Figure 6a shows the H_2_ production activity of MnO_2_-AC (0.1 g/L) as a function of the sulphide ion concentration. As the sulphide ion concentration increased from 0.05 to 0.2 M, the H_2_ production rate increased gradually. When the sulphide ion concentration exceeded 0.2 M, however, the hydrogen production rate decreased unexpectedly. Such a sudden decease of hydrogen production at the higher ion concentration can be explained by photocatalytic corrosion on the catalyst surface, which degrades the hydrogen production efficiency [65,66]. When using sulphite ion-involved wastewater, we observed a similar feature of hydrogen production activities (Figure 6b). Namely, the H_2_ production rate increased with increasing sulphite ion concentration up to 0.25 M, whereas that suddenly decreased at the high sulphite ion concentration of 0.30 M due to blocking of active sites by the penetrated sulphite ions [67,68].

Based upon the above results, we chose the optimal concentrations for sulphide (best value 0.25 M), and sulphite ions (best value 0.2 M), and mixed to those solutions. Then, the effect of the catalyst dosage on the hydrogen production was examined (Figure 6c). As the catalyst dose increased up to 0.25 g/L, the H_2_ production rate increased because of the increased number of active sites. For a higher dose of 0.30 g/L, the hydrogen production rate began to decrease because the high density of MnO_2_ nanocomposites may give rise to viscosity of the solution, and it will retard the photon collection at the photocatalyst surface and will degrade the oxidation-reduction reaction for H_2_ production [67,68,69]. Next, we tested the reusability of MnO_2_-AC as a photocatalytic H_2_ production agent for the sulphuric wastewater treatment. For this test, we chose the best values of experimental parameters obtained from the previous experiments (i.e., photocatalyst dose = 0.25 g/L, sulphide ion concentration = 0.2 M, sulphite ion concentration 0.25 M). In addition, we note that, after every cycle, the MnO_2_-AC catalyst was set for each measurement. For this step, the plain solution was separated, and the recovered photocatalyst was washed in DI water several times and reused for the next cycle. As shown in Figure 6d, the rate of hydrogen production gradually decreased as the cycle number increased. This can be interpreted by the catalyst loss of the trace quantity during every recycle [65,70] because the 30% weight loss of MnO_2_-AC was observed at the end of the fifth recycle.

**Figure 7:**
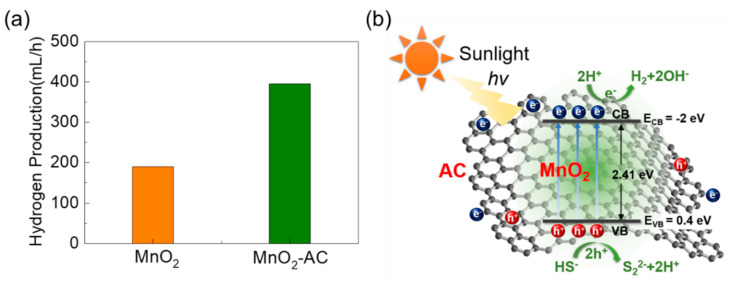
(**a**) Comparison of photocatalytic hydrogen production rates between MnO_2_ nanoparticles and MnO_2_-AC nanocomposites. (**b**) Photocatalytic hydrogen production mechanism in MnO_2_-AC nanocomposites.

Through mimicking the above experiments for MnO_2_, we obtained the maximum H_2_ production of 190 mL/h (Figure 7a). Therefore, we can conclude that the hydrogen production efficiency is much better for the MnO_2_-AC nanocomposites (395 mL/h) than the bare MnO_2_ nanoparticles, which is higher than the previous reports (Table S1). To help understand the superior H_2_ production activity in MnO_2_-AC, we explain the mechanism of photocatalytic hydrogen production using MnO_2_-AC. The valence and the conduction band edges can be found by using the following equations:

(1) $$E_{VB}=X-E_{e}+0.5E_{g}$$

(2) $$E_{CB}=E_{VB}-E_{g}$$

where E*_VB_* and E*_CB_* are the potential energy values of the valence band and the conduction band, respectively. X is the electronegativity of the semiconducting material, and E*_e_* is the free electron energy on the hydrogen scale (4.5 eV), and E*_g_* is the bandgap energy of the material. In this study, we observed MnO_2_-AC to possess E*_g_* of 2.41 eV (Figure 5b); hence, E*_VB_* and E*_CB_* can be 0.4 and −2 eV, respectively. Based on these values, one can illustrate the energy band diagram of the MnO_2_-AC composite system (Figure 7b). When irradiating sunlight onto the MnO_2_-AC nanocomposites, the electrons will jump from the valence band to the conduction band. Such an electron excitation will also create the photo-generated holes in the valence band. These photo-generated electrons and holes may cause H_2_ production with their chemical reactions with sulphuric wastewater as follows:

(3) $${\mathrm{MnO}}_{2}/\mathrm{AC}\overset{\mathrm{hv}}{\to }{\mathrm{MnO}}_{2}-\mathrm{AC}\left(e_{\mathrm{cb}}^{-}+h_{\mathrm{vb}}^{+}\right)$$

(4) $$h_{\mathrm{vb}}^{+}+{\mathrm{HS}}^{-}\to 2H^{+}+S_{2}^{2-}$$

(5) $$2e_{\mathrm{cb}}^{-}+2H^{+}\to H^{\cdot }+H^{\cdot }\to H_{2}$$

Namely, the hole carriers may react with the hydrogen sulphide ions, and produce the hydrogen ions. Then, the hydrogen ions will be eventually reduced into hydrogen molecules by reaction with photo-generated electrons. For this mechanism, H_2_ production will be much enhanced when the material system has a larger surface area (i.e., abundant active sites for chemical reaction) and a higher carrier conductivity (i.e., fast promotion of chemical reaction). As a consequance, the MnO_2_-AC nanocomposites with high porosity and high conductivity yield a higher H_2_ production rate, particularly, compared to bare MnO_2_.

## 4. Conclusions

The MnO_2_-AC nanocomposites were prepared by using the biomass resources of *Brassica oleracea* and *Azadirachta indica*. The MnO_2_-AC nanocomposites showed the aggregated structure of spherical α-MnO_2_ nanoparticles-decorated AC nanoflakes. When using the MnO_2_-AC nanocomposites as a photocatalytic agent for the sulphuric wastewater treatment, the enhanced hydrogen production rate of 395 mL/h was achieved. We attributed such an excellent H_2_ production activity to high conductivity and high porosity of the MnO_2_-AC composite system (i.e., fast promotion and abundant active sites for chemical reaction). The results suggest that the ecofriendly-synthesized MnO_2_-AC nanocomposite system is useful for upcycling of wastewater via photocatalytic hydrogen production.

## Supplementary Materials

The following are available online at https://www.mdpi.com/2079-4991/10/8/1610/s1, Figure S1: Schematic illustration of photocatalytic trapezoidal reactor; Figure S2: (a) Nitrogen adsorption–desorption isotherm characteristics and (b) Pore distribution properties of activated carbon; Table S1: Comparison of hydrogen production activity of MnO2 and MnO2-AC with other oxide-based photocatalysts reported in previous studies.

## References

[1] Tsuji I., Kato H., Kudo A. (2005). Visible-Light-Induced H_2_ Evolution from an Aqueous Solution Containing Sulfide and Sulfite over a ZnS–CuInS_2_–AgInS_2_ Solid-Solution Photocatalyst. Angew. Chem. Int. Ed..

[2] Das S., Samanta A., Jana S. (2017). Light-Assisted Synthesis of Hierarchical Flower-Like MnO_2_ Nanocomposites with Solar Light Induced Enhanced Photocatalytic Activity. ACS Sustain. Chem. Eng..

[3] Preethi V., Kanmani S. (2014). Photocatalytic hydrogen production using Fe_2_O_3_-based core shell nano particles with ZnS and CdS. Int. J. Hydrogen Energy.

[4] Vikrant K., Kim K.-H., Deep A. (2019). Photocatalytic mineralization of hydrogen sulfide as a dual-phase technique for hydrogen production and environmental remediation. Appl. Catal. B.

[5] Kida T., Guan G., Yamada N., Ma T., Kimura K., Yoshida A. (2004). Hydrogen production from sewage sludge solubilized in hot-compressed water using photocatalyst under light irradiation. Int. J. Hydrogen Energy.

[6] Moon S.Y., Gwag E.H., Park J.Y. (2018). Hydrogen Generation on Metal/Mesoporous Oxides: The Effects of Hierarchical Structure, Doping, and Co-catalysts. Energy Technol..

[7] Concina I., Ibupoto Z.H., Vomiero A. (2017). Semiconducting Metal Oxide Nanostructures for Water Splitting and Photovoltaics. Adv. Energy Mater..

[8] Huang J., Li X., Jin X., Wang L., Deng Y., Su F., Wong P.K., Ye L. (2020). High-efficiency and stable photocatalytic hydrogen evolution of rhenium sulfide co-catalyst on Zn_0.3_Cd_0.7_S. Mater. Adv..

[9] Djurišić A.B., Leung Y.H., Ching Ng A.M. (2014). Strategies for improving the efficiency of semiconductor metal oxide photocatalysis. Mater. Horiz..

[10] Liu J., Ge X., Ye X., Wang G., Zhang H., Zhou H., Zhang Y., Zhao H. (2016). 3D graphene/δ-MnO_2_ aerogels for highly efficient and reversible removal of heavy metal ions. J. Mater. Chem. A.

[11] Crespo Y., Seriani N. (2014). A lithium peroxide precursor on the α-MnO_2_ (100) surface. J. Mater. Chem. A.

[12] Zhang K., Han X., Hu Z., Zhang X., Tao Z., Chen J. (2015). Nanostructured Mn-based oxides for electrochemical energy storage and conversion. Chem. Soc. Rev..

[13] Truong T.T., Liu Y., Ren Y., Trahey L., Sun Y. (2012). Morphological and Crystalline Evolution of Nanostructured MnO_2_ and Its Application in Lithium–Air Batteries. ACS Nano.

[14] Debnath B., Roy A.S., Kapri S., Bhattacharyya S. (2016). Efficient Dye Degradation Catalyzed by Manganese Oxide Nanoparticles and the Role of Cation Valence. ChemistrySelect.

[15] Rahmat M., Rehman A., Rahmat S., Bhatti H.N., Iqbal M., Khan W.S., Bajwa S.Z., Rahmat R., Nazir A. (2019). Highly efficient removal of crystal violet dye from water by MnO_2_ based nanofibrous mesh/photocatalytic process. J. Mater. Res. Technol..

[16] Vidya Lekshmi K.P., Yesodharan S., Yesodharan E.P. (2018). MnO_2_ efficiently removes indigo carmine dyes from polluted water. Heliyon.

[17] Husnain S.M., Asim U., Yaqub A., Shahzad F., Abbas N. (2020). Recent trends of MnO_2_-derived adsorbents for water treatment: A review. New J. Chem..

[18] Wang R., Hao Q., Feng J., Wang G.-C., Ding H., Chen D., Ni B. (2019). Enhanced separation of photogenerated charge carriers and catalytic properties of ZnO-MnO_2_ composites by microwave and photothermal effect. J. Alloys Compd..

[19] Chhabra T., Kumar A., Bahuguna A., Krishnan V. (2019). Reduced graphene oxide supported MnO_2_ nanorods as recyclable and efficient adsorptive photocatalysts for pollutants removal. Vacuum.

[20] Zhao J., Zhao Z., Li N., Nan J., Yu R., Du J. (2018). Visible-light-driven photocatalytic degradation of ciprofloxacin by a ternary Mn_2_O_3_/Mn_3_O_4_/MnO_2_ valence state heterojunction. Chem. Eng. J..

[21] Rajrana K., Gupta A., Mir R.A., Pandey O.P. (2019). Facile sono-chemical synthesis of nanocrystalline MnO_2_ for catalytic and capacitive applications. Phys. B.

[22] Chiam S.-L., Pung S.-Y., Yeoh F.-Y. (2020). Recent developments in MnO_2_-based photocatalysts for organic dye removal: A review. Environ. Sci. Pollut. Res..

[23] Sankar S., Sharma S.K., An N., Lee H., Kim D.Y., Im Y.B., Cho Y.D., Ganesh R.S., Ponnusamy S., Raji P. (2016). Photocatalytic properties of Mn-doped NiO spherical nanoparticles synthesized from sol-gel method. Optik.

[24] Ding Y., Wei D., He R., Yuan R., Xie T., Li Z. (2019). Rational design of Z-scheme PtS-ZnIn_2_S_4_/WO_3_-MnO_2_ for overall photo-catalytic water splitting under visible light. Appl. Catal. B.

[25] Zhen W., Ning X., Wang M., Wu Y., Lu G. (2018). Enhancing hydrogen generation via fabricating peroxide decomposition layer over NiSe/MnO_2_-CdS catalyst. J. Catal..

[26] Abuzeid H.M., Elsherif S.A., Abdel Ghany N.A., Hashem A.M. (2019). Facile, cost-effective and eco-friendly green synthesis method of MnO_2_ as storage electrode materials for supercapacitors. J. Energy Storage.

[27] Hashem A.M., Abuzeid H.M., Winter M., Li J., Julien C.M. (2020). Synthesis of High Surface Area α-KyMnO_2_ Nanoneedles Using Extract of Broccoli as Bioactive Reducing Agent and Application in Lithium Battery. Materials.

[28] Zia J., Aazam E.S., Riaz U. (2020). Synthesis of nanohybrids of polycarbazole with α-MnO_2_ derived from Brassica oleracea: A comparison of photocatalytic degradation of an antibiotic drug under microwave and UV irradiation. Environ. Sci. Pollut. Res..

[29] Gawande M.B., Goswami A., Felpin F.-X., Asefa T., Huang X., Silva R., Zou X., Zboril R., Varma R.S. (2016). Cu and Cu-Based Nanoparticles: Synthesis and Applications in Catalysis. Chem. Rev..

[30] Sunkar S., Nachiyar C.V. (2012). Biogenesis of antibacterial silver nanoparticles using the endophytic bacterium Bacillus cereus isolated from Garcinia xanthochymus. Asian Pac. J. Trop Biomed..

[31] Sanchez-Botero L., Herrera A.P., Hinestroza J.P. (2017). Oriented Growth of α-MnO_2_ Nanorods Using Natural Extracts from Grape Stems and Apple Peels. Nanomaterials.

[32] Guo T., Yao M.-S., Lin Y.-H., Nan C.-W. (2015). A comprehensive review on synthesis methods for transition-metal oxide nanostructures. CrystEngComm.

[33] Chan Y.L., Pung S.Y., Hussain N.S., Sreekantan S., Yeoh F.Y. (2013). Photocatalytic Degradation of Rhodamine B Using MnO_2_ and ZnO Nanoparticles. Mater. Sci. Forum.

[34] Zhang G., Qu J., Du Y., Guo F., Zhao H., Zhang Y., Xu Y. (2014). Hydrogen production from CO_2_ reforming of methane over high pressure H_2_O_2_ modified different semi-cokes. J. Ind. Eng. Chem..

[35] Zhang G., Dong Y., Feng M., Zhang Y., Zhao W., Cao H. (2010). CO_2_ reforming of CH_4_ in coke oven gas to syngas over coal char catalyst. Chem. Eng. J..

[36] Sekar S., Aqueel Ahmed A.T., Pawar S.M., Lee Y., Im H., Kim D.Y., Lee S. (2020). Enhanced water splitting performance of biomass activated carbon-anchored WO_3_ nanoflakes. Appl. Surf. Sci..

[37] Sankar S., Ahmed A.T.A., Inamdar A.I., Im H., Im Y.B., Lee Y., Kim D.Y., Lee S. (2019). Biomass-derived ultrathin mesoporous graphitic carbon nanoflakes as stable electrode material for high-performance supercapacitors. Mater. Des..

[38] Sekar S., Lee Y., Kim D.Y., Lee S. (2019). Substantial LIB anode performance of graphitic carbon nanoflakes derived from biomass green-tea waste. Nanomaterials.

[39] Sankar S., Inamdar A.I., Im H., Lee S., Kim D.Y. (2018). Template-free rapid sonochemical synthesis of spherical α-MnO_2_ nanoparticles for high-energy supercapacitor electrode. Ceram. Int..

[40] Hashem A.M., Abuzeid H., Kaus M., Indris S., Ehrenberg H., Mauger A., Julien C.M. (2018). Green synthesis of nanosized manganese dioxide as positive electrode for lithium-ion batteries using lemon juice and citrus peel. Electrochim. Acta.

[41] Fang S., Lin F., Qu D., Liang X., Wang L. (2019). Characterization of Purified Red Cabbage Anthocyanins: Improvement in HPLC Separation and Protective Effect against H_2_O_2_-Induced Oxidative Stress in HepG_2_ Cells. Molecules.

[42] Zhang Y., Gong X., Zhang B., Liu W., Xu M. (2014). Potassium catalytic hydrogen production in sorption enhanced gasification of biomass with steam. Int. J. Hydrogen Energy.

[43] Bulushev D.A., Jia L., Beloshapkin S., Ross J.R.H. (2012). Improved hydrogen production from formic acid on a Pd/C catalyst doped by potassium. Chem. Commun..

[44] Balakumar V., Ryu J.W., Kim H., Manivannan R., Son Y.-A. (2020). Ultrasonic synthesis of α-MnO_2_ nanorods: An efficient catalytic conversion of refractory pollutant, methylene blue. Ultrason. Sonochem..

[45] Sekar S., Kim D.Y., Lee S. (2020). Excellent Oxygen Evolution Reaction of Activated Carbon-Anchored NiO Nanotablets Prepared by Green Routes. Nanomaterials.

[46] Sankar S., Saravanan S., Ahmed A.T.A., Inamdar A.I., Im H., Lee S., Kim D.Y. (2019). Spherical activated-carbon nanoparticles derived from biomass green tea wastes for anode material of lithium-ion battery. Mater. Lett..

[47] Lee S., Kang T.W., Kim D.Y. (2005). Correlation of Magnetic Properties with Microstructural Properties for Columnar-Structured (Zn_1−x_Mn_x_)O/Al_2_O_3_ (0001) Thin Films. J. Cryst. Growth.

[48] Kaur N., Lee Y., Kim D.Y., Lee S. (2018). Optical bandgap tuning in nanocrystalline ZnO:Y films via forming defect-induced localized bands. Mater. Des..

[49] Lee S., Seong J., Kim D. (2010). Effects of laser-annealing using a KrF excimer laser on the surface, structural, optical, and electrical properties of AlZnO thin films. J. Korean Phys. Soc..

[50] Ali G.A.M., Yusoff M.M., Shaaban E.R., Chong K.F. (2017). High performance MnO_2_ nanoflower supercapacitor electrode by electrochemical recycling of spent batteries. Ceram. Int..

[51] Wang J.-W., Chen Y., Chen B.-Z. (2015). A Synthesis Method of MnO2/Activated Carbon Composite for Electrochemical Supercapacitors. J. Electrochem. Soc..

[52] Liu R., Liu E., Ding R., Liu K., Teng Y., Luo Z., Li Z., Hu T., Liu T. (2015). Facile in-situ redox synthesis of hierarchical porous activated carbon@MnO_2_ core/shell nanocomposite for supercapacitors. Ceram. Int..

[53] Kumar A., Aathira M.S., Pal U., Jain S.L. (2018). Photochemical Oxidative Coupling of 2-Naphthols using a Hybrid Reduced Graphene Oxide/Manganese Dioxide Nanocomposite under Visible-Light Irradiation. ChemCatChem.

[54] Darari A., Ardiansah H.R., Arifin Rismaningsih N., Ningrum A.N., Subagio A. (2016). Characterization of CNT-MnO_2_ nanocomposite by electrophoretic deposition as potential electrode for supercapacitor. AIP Conf. Proc..

[55] Jamesh M.-I. (2019). Recent advances on flexible electrodes for Na-ion batteries and Li–S batteries. J. Energy Chem..

[56] Zhang G., Ren L., Hu D., Gu H., Zhang S. (2018). Sulfuric acid etching for fabrication of porous MnO_2_ for high-performance supercapacitor. J. Colloid Interface Sci..

[57] Abdel-Aal S.K., Aly A.E., Chanduví H.H.M., Gil Rebaza A.V., Atteia E., Shankar A. (2020). Magnetic and optical properties of perovskite-graphene nanocomposites LaFeO_3_-rGO: Experimental and DFT calculations. Chem. Phys..

[58] Ateia E.E., Mohamed A.T. (2017). Nonstoichiometry and phase stability of Al and Cr substituted Mg ferrite nanoparticles synthesized by citrate method. J. Magn. Magn. Mater..

[59] Lee S., Lee Y., Kim D.Y., Panin G.N. (2016). Multicolor Emission from Poly(p-Phenylene)/Nanoporous ZnMnO Organic–Inorganic Hybrid Light-Emitting Diode. ACS Appl. Mater. Interfaces.

[60] Lee S., Lee Y., Panin G.N. (2017). Novel Green Luminescent and Phosphorescent Material: Semiconductive Nanoporous ZnMnO with Photon Confinement. ACS Appl. Mater. Interfaces.

[61] Lee S., Lee Y., Kim D.Y., Panin G.N. (2019). Highly Efficient Low-Voltage Cathodoluminescence of Semiconductive Nanoporous ZnMnO Green Phosphor Films. Appl. Surf. Sci..

[62] Kamran M.A. (2018). The aggregation of Mn^2+^, its d-d transition in CdS:Mn(II) nanobelts and bound magnetic polaron formation at room temperature. Nanotechnology.

[63] Liu Q., Sun Z., Yan W., Zhong W., Pan Z., Hao L., Wei S. (2007). Anomalous magnetic behavior of Mn-Mn dimers in the dilute magnetic semiconductor (Ga,Mn)N. Phys. Rev. B.

[64] Li T., Wu J., Xiao X., Zhang B., Hu Z., Zhou J., Yang P., Chen X., Wang B., Huang L. (2016). Band gap engineering of MnO_2_ through in situ Al-doping for applicable pseudocapacitors. RSC Adv..

[65] Navakoteswara Rao V., Lakshmana Reddy N., Mamatha Kumari M., Ravi P., Sathish M., Kuruvilla K.M., Preethi V., Reddy K.R., Shetti N.P., Aminabhavi T.M. (2019). Photocatalytic recovery of H_2_ from H_2_S containing wastewater: Surface and interface control of photo-excitons in Cu_2_S@TiO_2_ core-shell nanostructures. Appl. Catal. B.

[66] Jayabalan T., Matheswaran M., Preethi V., Naina Mohamed S. (2020). Enhancing biohydrogen production from sugar industry wastewater using metal oxide/graphene nanocomposite catalysts in microbial electrolysis cell. Int. J. Hydrogen Energy.

[67] Anthony Raja M., Preethi V. (2020). Photocatalytic hydrogen production using bench-scale trapezoidal photocatalytic reactor. Int. J. Hydrogen Energy.

[68] Anthony Raja M., Preethi V. (2020). Performance of Square and Trapezoidal photoreactors for solar hydrogen recovery from various industrial sulphide wastewater using CNT/Ce^3+^ doped TiO_2_. Int. J. Hydrogen Energy.

[69] Li P., Chen R., Tian S., Xiong Y. (2019). Efficient Oxygen Evolution Catalysis Triggered by Nickel Phosphide Nanoparticles Compositing with Reduced Graphene Oxide with Controlled Architecture. ACS Sustain. Chem. Eng..

[70] Luo Z., Zhao X., Zhang H., Jiang Y. (2019). Zn_0.3_Cd_0.7_S nanorods loaded with noble-metal-free Ni_3_C co-catalyst enhancing photocatalytic hydrogen evolution. Appl. Catal. A.

