# Supplementary material for: Upcycling of Wastewater via Effective Photocatalytic Hydrogen Production Using MnO2 Nanoparticles—Decorated Activated Carbon Nanoflakes
Source: Nanomaterials (Basel). 2020 Aug 17;10(8):1610. doi: 10.3390/nano10081610 (PMC7466657; doi:10.3390/nano10081610)
Supplement: Supplementary file 1 [file nanomaterials-10-01610-s001.pdf]

nanomaterials

Supplementary Materials

*Article*

# Upcycling of Wastewater via Effective Photocatalytic Hydrogen Production Using $MnO_2$ Nanoparticles—Decorated Activated Carbon Nanoflakes

**Sankar Sekar [1,2], Sejoon Lee [1,2,*], Preethi Vijayarengan [3,*], Kaliyappan Mohan Kalirajan [4], Thirumavalavan Santhakumar [3], Saravanan Sekar [5], and Sutha Sadhasivam [6]**

1 Division of Physics & Semiconductor Science, Dongguk University-Seoul, Seoul 04620, Republic of Korea
2 Quantum-functional Semiconductor Research Center, Dongguk University-Seoul, Seoul 04620, Republic of Korea
3 Renewable Energy Lab, Hindustan Institute of Technology and Science, Chennai-603103, Tamil Nadu, India
4 Department of Nanotechnology, K.S.R College of Technology, Tiruchengode-637215, Tamil Nadu, India
5 Department of Mechanical Engineering, K. Ramakrishnan College of Technology, Trichy-621112, Tamil Nadu, India
6 Department of chemistry, Periyar University, Salem-636011, Tamil Nadu, India

* Correspondence: sejoon@dongguk.edu (S. Lee); preethi_enviro@hotmail.com (V. Preethi)

## ■ Photocatalytic Trapezoidal Reactor

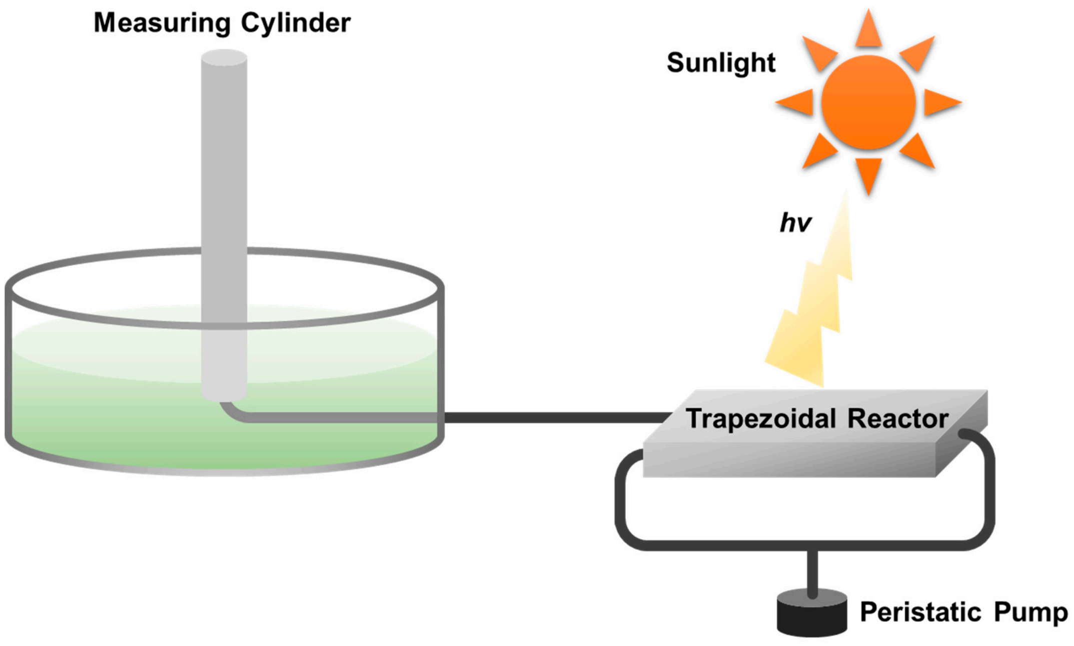


**Figure S1.** Schematic illustration of photocatalytic trapezoidal reactor.

## ■ Textural Characteristics Biomass Activated Carbon

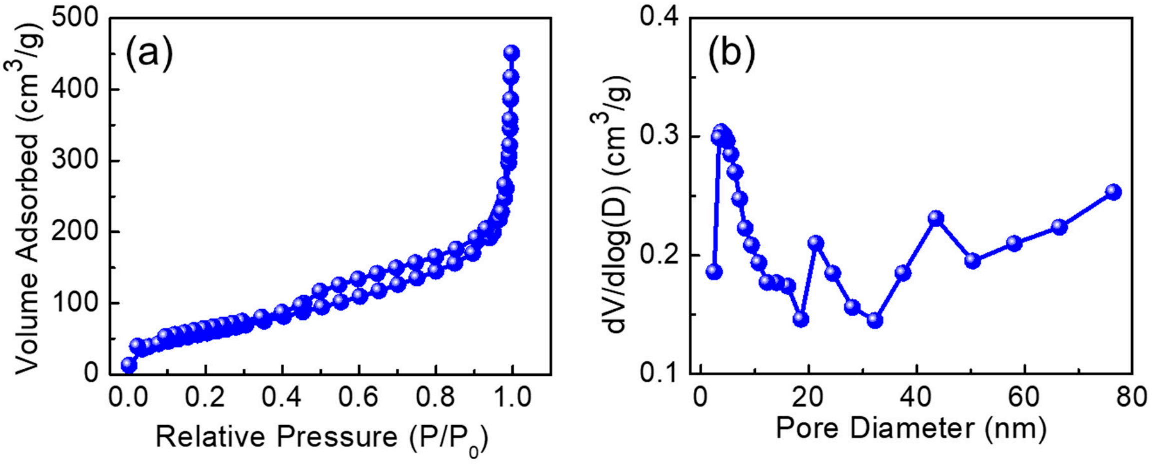


**Figure S2.** (**a**) Nitrogen adsorption–desorption isotherm characteristics and (**b**) Pore distribution properties of activated carbon.

**Table S1.** Comparison of hydrogen production activity of $MnO_2$ and $MnO_2$-AC with other oxide-based photocatalysts reported in previous studies.

| Catalyst | Light Source | Hydrogen Production (mL/h/g) | Reference |
|---|---|---|---|
| **$MnO_2$-AC** | Solar light | 395 | This work |
| **$MnO_2$** | Solar light | 190 | This work |
| **NiSe/$MnO_2$-CdS** | Xenon lamp | 10 | [1] |
| **PtS-$ZnIn_2S_4$/ $WO_3$-$MnO_2$** | UV lamp | 142 | [2] |
| **CNT- CdZnS/$Fe_2O_3$** | Solar light | 450 | [3] |
| **$Cu_2S$@$TiO_2$** | UV lamp | 41.6 | [4] |
| **$TiO_2$/$Fe_2O_3$** | UV lamp | 2700 μ | [5] |

## References


1. Zhen, W.; Ning, X.; Wang, M.; Wu, Y.; Lu, G., Enhancing hydrogen generation via fabricating peroxide decomposition layer over NiSe/$MnO_2$-CdS catalyst. *J. Catal.* **2018,** *367*, 269-282.s
2. Ding, Y.; Wei, D.; He, R.; Yuan, R.; Xie, T.; Li, Z., Rational design of Z-scheme PtS-$ZnIn_2S_4$/$WO_3$-$MnO_2$ for overall photo-catalytic water splitting under visible light. *Appl. Catal. B* **2019,** *258*, 117948.
3. Anthony Raja, M.; Preethi, V., Photocatalytic hydrogen production using bench-scale trapezoidal photocatalytic reactor. *Int. J. Hydrog. Energy* **2020,** *45*, 7574-7583.
4. Navakoteswara Rao, V.; Lakshmana Reddy, N.; Mamatha Kumari, M.; Ravi, P.; Sathish, M.; Kuruvilla, K. M.; Preethi, V.; Reddy, K. R.; Shetti, N. P.; Aminabhavi, T. M.; Shankar, M. V., Photocatalytic recovery of $H_2$ from $H_2S$ containing wastewater: Surface and interface control of photo-excitons in $Cu_2S$@$TiO_2$ core-shell nanostructures. *Appl. Catal. B* **2019,** *254*, 174-185.
5. Madhumitha, A.; Preethi, V.; Kanmani, S., Photocatalytic hydrogen production using $TiO_2$ coated iron-oxide core shell particles. *Int. J. Hydrog. Energy* **2018,** *43*, 3946-3956.
